# Individual factors associated with suicidal recurrence in patients of southern tunisia

**DOI:** 10.1192/j.eurpsy.2021.1570

**Published:** 2021-08-13

**Authors:** M. Tfifha, W. Abbes, W. Mahdhaoui, A. Frikha, L. Ghanmi

**Affiliations:** 1 Department Of Psychiatry, regional hospital of gabes, gabes, Tunisia; 2 The Department Of Psychiatry, Hospital of gabes, Gabes, Tunisia

**Keywords:** suicidal attempts, suicidal recurrence, individual factors, Suicide prevention

## Abstract

**Introduction:**

Nowadays, suicide is a global public health problem thus detection of risk factors more specifically individual factors can be used as a method for prevention and intervention.

**Objectives:**

The aims of our study were to assess the incidence of suicidal recurrence and its individual associated factors.

**Methods:**

A retrospective descriptive and analytical study was undertaken including all patients consulting for the first time at Gabes psychiatry department (in southern Tunisia) from the 4^th^ March 2009 to the 25^th^ September 2020 for suicidal attempt. Sociodemographic and clinical data as well as suicidal attempts’ characteristics were assessed. The statistical analysis was executed on the software SPSS (20^th^ edition).

**Results:**

278 patients were collected including 217 female. The mean age was 26. Suicidal patients were unmarried (75.9%), childless (79.1%) and unemployed (47.5%). The common suicidal attempt method was voluntary drug intoxication (67.8%). Interference of individual factors was reported in 77% of cases, especially difficulties to cope with stress (46.4%), followed by low self-esteem (36.5%), personal psychiatric history (17.3%), personal medical history (8.3%) and alcohol or drug abuse (6.1%). A suicidal re-attempt was notedin 24.9 % of cases. Recurrence was associated with the female gender (72.4%), difficulties to cope with stress (<10^-3^) and low self-esteem (p=0.012).

**Conclusions:**

After the first suicidal attempt, it’s crucial to identify the individual factors that seems to have an influence on subsequent suicidal behaviour in order to ensure a proper treatment.

